# Using the Multiphase Optimization Strategy (MOST) framework to test intervention delivery strategies: a study protocol

**DOI:** 10.1186/s13063-019-3853-y

**Published:** 2019-12-16

**Authors:** Sarabeth Broder-Fingert, Jocelyn Kuhn, Radley Christopher Sheldrick, Andrea Chu, Lisa Fortuna, Megan Jordan, Dana Rubin, Emily Feinberg

**Affiliations:** 1 Boston Medical Center, 801 Albany Street, Boston, MA 02114 USA; 2 Boston University School of Medicine, Boston, MA USA; 3 Boston University School of Public Health, Boston, MA USA; 4 DotHouse Health Center, Dorchester, MA USA

**Keywords:** Family Navigation, Multiphase Optimization Strategy, Child behavioral health services, Health disparities

## Abstract

**Background:**

Delivery of behavioral interventions is complex, as the majority of interventions consist of multiple components used either simultaneously, sequentially, or both. The importance of clearly delineating delivery strategies within these complex interventions—and furthermore understanding the impact of each strategy on effectiveness—has recently emerged as an important facet of intervention research. Yet, few methodologies exist to prospectively test the effectiveness of delivery strategies and how they impact implementation. In the current paper, we describe a study protocol for a large randomized controlled trial in which we will use the Multiphase Optimization Strategy (MOST), a novel framework developed to optimize interventions, i.e., to test the effectiveness of intervention delivery strategies using a factorial design. We apply this framework to delivery of Family Navigation (FN), an evidence-based care management strategy designed to reduce disparities and improve access to behavioral health services, and test four components related to its implementation.

**Methods/design:**

The MOST framework contains three distinct phases: Preparation, Optimization, and Evaluation. The Preparation phase for this study occurred previously. The current study consists of the Optimization and Evaluation phases. Children aged 3-to-12 years old who are detected as “at-risk” for behavioral health disorders (*n* = 304) at a large, urban federally qualified community health center will be referred to a Family Partner—a bicultural, bilingual member of the community with training in behavioral health and systems navigation—who will perform FN. Families will then be randomized to one of 16 possible combinations of FN delivery strategies (2 × 2 × 2× 2 factorial design). The primary outcome measure will be achieving a family-centered goal related to behavioral health services within 90 days of randomization. Implementation data on the fidelity, acceptability, feasibility, and cost of each strategy will also be collected. Results from the primary and secondary outcomes will be reviewed by our team of stakeholders to optimize FN delivery for implementation and dissemination based on effectiveness, efficiency, and cost.

**Discussion:**

In this protocol paper, we describe how the MOST framework can be used to improve intervention delivery. These methods will be useful for future studies testing intervention delivery strategies and their impact on implementation.

**Trial registration:**

ClinicalTrials.gov, NCT03569449. Registered on 26 June 2018.

## Contributions to the literature

This study offers the following additions to the literature:

- Traditional two-arm randomized controlled trials are limited in that they provide information about the general effectiveness of a packaged intervention. In contrast, the Multiphase Optimization Strategy (MOST) framework requires analysis of components and determines the optimized version of an intervention.
- The MOST framework is particularly relevant to scaled implementation of Family Navigation (FN) interventions because of overall concerns related to cost, efficiency, and effectiveness of this intervention across contexts.
- This study protocol offers an innovative application of the MOST framework to improve the delivery of FN, which is an intervention that aims to improve accessibility of health services to historically underserved populations.

## Background

### Intervention optimization

Delivery of behavioral interventions can be complex, as the majority of interventions consist of multiple components that can differ by order (e.g., simultaneously, sequentially, or both), locations (e.g., home, medical setting), method (e.g., in-person, remote), or individual (i.e., teacher, therapist). The importance of developing and testing efficient, effective delivery strategies for complex interventions has recently emerged as an important facet of intervention research [1]. Yet, few methodologies allow for both the rigor of a randomized clinical trial and the flexibility and adaptability of designing and testing delivery for scale. This mismatch (i.e., the narrow focus of a randomized trial in which one or two strategies can be compared with the need for testing multiple delivery strategies) has contributed to a catalog of interventions that are “evidence-based,” yet without efficient strategies for delivery.

In 2005, Collins and colleagues developed the MOST framework [2], a guide for intervention developers that draws heavily from the fields of engineering, statistics, biostatistics, and behavioral science [3]. MOST involves three phases: Preparation, Optimization, and Evaluation. The Preparation phase consists of developing a conceptual model for the intervention; pilot testing; identifying “core components”; and determining what outcomes should be optimized (e.g., effectiveness, efficiency, cost). The Optimization phase uses a multifactorial design to conduct a randomized factorial experiment of specific components identified during the Preparation phase. Finally, the Evaluation phase consists of reviewing results of the trial and developing consensus regarding intervention components. Since its initial publication [4], multiple studies have utilized MOST to develop and test intervention components [5–10] with a primary focus on effectiveness [4, 10, 11].

### Optimizing intervention delivery for scale: Family Navigation as an exemplar

Another promising use of the MOST framework is optimizing intervention delivery. Many interventions—particularly complex interventions—have components that are fixed, but require a variety of delivery strategies for patients or clients that are efficient and effective [1]. FN is an example of a complex, evidence-based intervention. FN is a care management strategy designed to reduce disparities in care [12]. Traditional models utilize trained community health workers who assist families in overcoming systems and patient barriers to services over a time-limited period. FN is rooted in the chronic care model [13] and has evidence in multiple diseases as a means to reduce disparities by shortening the interval between discovery of risk (e.g., a positive screening mammogram for breast cancer) and diagnostic ascertainment [14–25].

Despite the promise of FN, studies demonstrate varying success upon translation from controlled research to real-world practice [26–30]. FN is a complex, multicomponent intervention which incorporates motivational interviewing (MI), problem-solving, education, and care coordination [29, 31]. FN can be delivered through a range of strategies: clinic-based meetings, home visits, or telehealth. FN delivery can be costly and time-consuming [32]. Learning how to optimize FN delivery by determining which strategies are most effective and efficient is critical to scalability and sustainability. At the same time, understanding FN’s cost as well as who benefits most is critical to decisions about how to optimally deploy available resources and generate the most cost-effective, equitable benefit.

### Frameworks

This study will rely on two frameworks, MOST and the Consolidated Framework for Implementation Research (CFIR) [33]. MOST was created as a means for developing “better interventions” by comparing components with the goal of optimization. In the current paper, we describe our protocol in which we use MOST to identify the most effective delivery package of FN. Use of this novel framework and study design offers the opportunity to optimize FN delivery, using empirical data, to support dissemination. Our primary objective is to compare how four different FN delivery strategies impact FN’s ability to enhance access to behavioral health services. Strategies we will compare include (1) enhanced care coordination technology vs. usual care, (2) community/home-based delivery vs. clinic-based delivery, (3) intensive symptom tracking vs. usual symptom tracking, and (4) individually tailored vs. structured, schedule-based visits. Outcomes of interest are access to behavioral health services. We will estimate the impact of the four delivery strategies on this primary outcome, and perform exploratory analyses regarding interactions between delivery conditions and interactions with patient characteristics. We will also assess implementation outcomes of fidelity, feasibility, acceptability, and cost quantitatively. Then, we will conduct semi-structured qualitative interviews based on the CFIR to further explore these implementation constructs. Finally, using consensus methods, we will combine data to develop an optimized delivery strategy.

## Methodology/design

### Overview

We will use a randomized, multifactorial design to simultaneously test four FN delivery strategies, as well as combinations of the strategies, ranging from the most basic (core FN) to the most intensive (enhanced care coordination technology + community visits + enhanced symptom monitoring + structured schedule-based visits). We will evaluate which combinations of delivery strategies are most effective and efficient in regard to the primary outcome (accessing behavioral health services). Stakeholders will then evaluate data to develop an optimized model of FN delivery. The study received approval from the Boston University Institutional Review Board (Protocol Number H-37634; ClinicalTrials.gov, NCT0356944) (Additional files 1 and 2).

### Setting

The study setting is a federally qualified community health center in a diverse, urban neighborhood in Boston, Massachusetts that serves > 3000 children per year in the study’s target age of 3–12 years old. Approximately 85% of patients are from a racial/ethnic minority group with > 80% using Medicaid. In addition to medical services, the health center provides comprehensive child behavioral health services. Multilingual social workers, licensed mental health clinicians, and a psychiatrist provide behavioral care in an onsite behavioral health department, and behavioral health clinicians integrated within primary care provide assessment and brief intervention.

### Participants

**Fig. 1:**
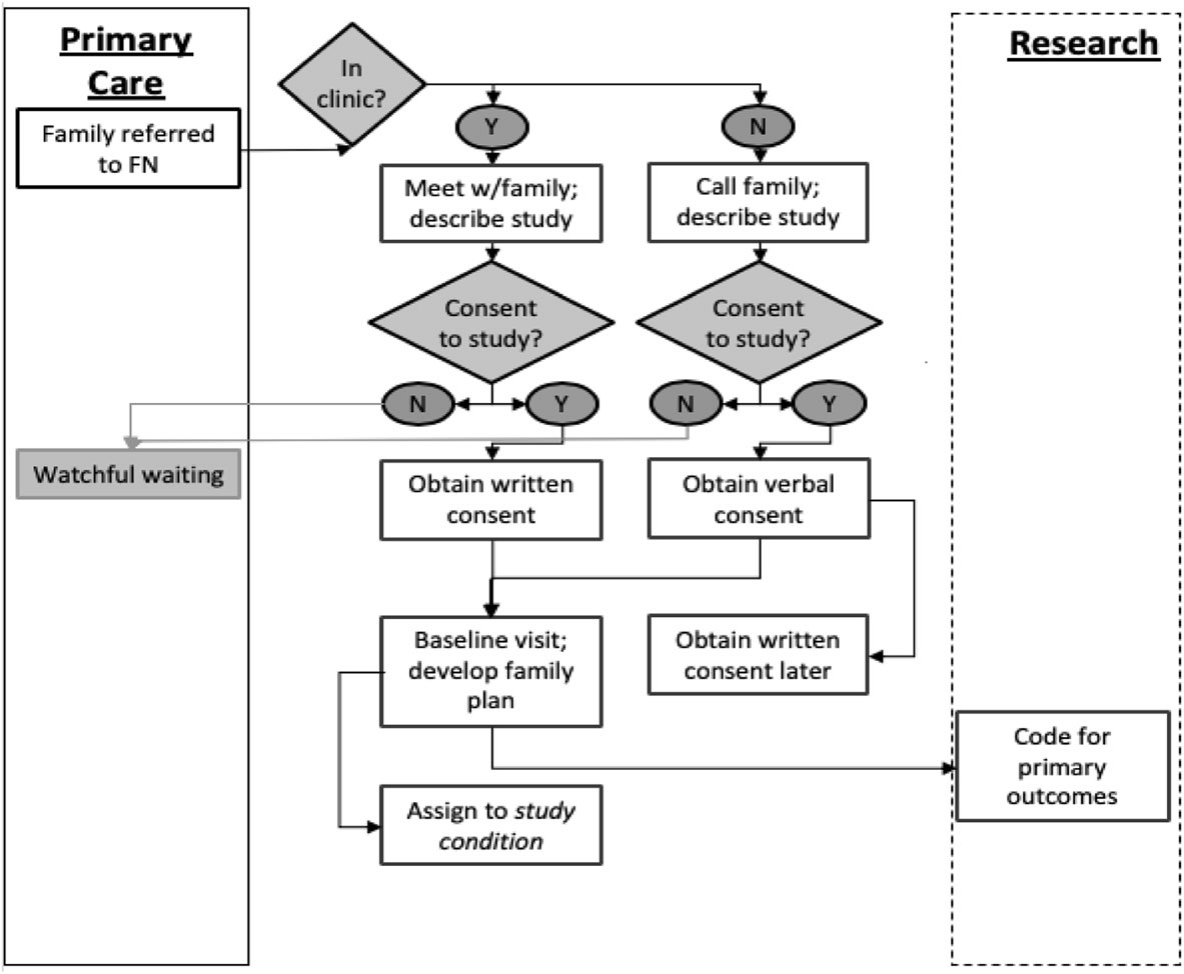
Recruitment process map

We will enroll 304 children and their families to be randomized to a combination of four delivery strategies (see Fig. 1). All children seen at the study site are screened for behavioral health concerns at all well-child visits or when parents raise behavioral concerns. For children aged 3 to 5 years, the Preschool Pediatric Symptom Checklist (PPSC) [34], which is part of the Survey of Well-being of Young Children (SWYC) [35], will be used. For those aged 6 to 12years old, the Pediatric Symptom Checklist-17 (PSC-17) [36–39] will be administered. If agreeable, families of children identified with a behavioral health concern will be referred to the study.

To promote enrollment and retention of participants, we will enroll families regardless of language and provide bicultural and bilingual services in Spanish and Vietnamese. Our Navigators—in this study called Family Partners (FPs)—are bilingual in either English/Spanish or English/Vietnamese, the most commonly spoken languages at the health center. Both the PSC-17 and SWYC are available in multiple languages. We will use telephonic translation services as needed. To promote collection of data across all study time points, FPs will be available to help families complete questionnaires in person, over the phone, or electronically. Families will receive weekly reminders when questionnaires are due.

We will also include a “watchful waiting” group for families of referred children who are not interested in accessing child behavioral services. The FP will ask parents if she can reach out again after 3 months to see if they desire services for the child at that time. Upon her recontacting the family, they will have the opportunity to enroll in the study if they would like services and wish to work with the FP.

### Core Family Navigation intervention

**Fig. 2:**
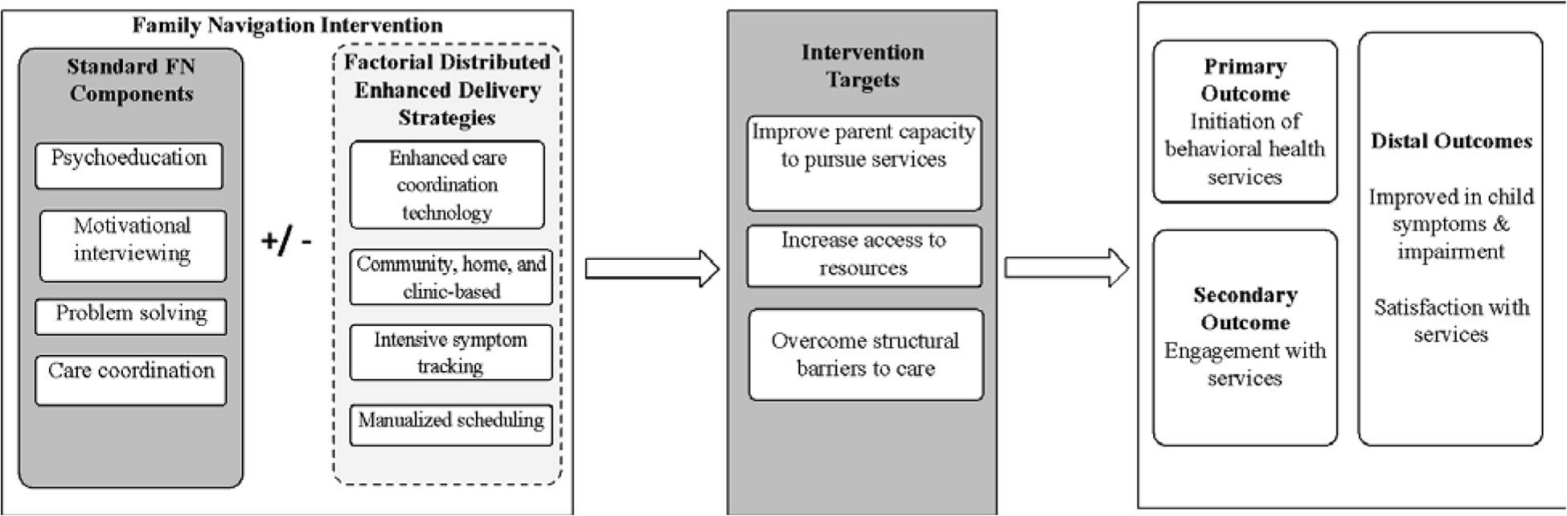
Mechanisms of Family Navigation

The core components of FN [12] will be delivered by an FP, a paraprofessional community member trained to support families of children with behavioral health needs. The FN mechanisms are shown in Fig. 2. Study procedures are designed to align with existing health center workflows. Participants will be allowed to receive concomitant care of any kind while participating in the trial. All families receive the following:

1. *Universal screening and behavioral health referral*. FN begins with a response to a positive behavioral health screening or parent concern for behavioral health issues. The FP will provide psychoeducation and use MI to explore family preferences regarding further evaluation, and referral to behavioral health services.
2. *Supporting access to behavioral health services*. The FP will work with the family to access recommended services, support family preferences, and engage in treatment through the creation of a Family Plan, which includes setting family-centered goals.
3. *Engagement in evidence-based treatment*. FN aims to support adherence to recommendation for behavioral healthcare. The FP, who is trained in MI and collaborative decision-making, will use these skills to support parental engagement in the behavioral health treatment plan.
4. *Monitoring to achieve family goals*. The FP continues until the goals articulated in the Family Plan are achieved, at which point the FP will be available as needed for up to 6 months.
5. *Family strengthening*. FPs will refer families to local support groups and parent mental health services if needed.
6. *Connection to concrete resources*. FPs receive extensive training on available local resources and connect families to community-based resources (e.g., disability insurance).

### Family contact with FP

#### First contact

Initial contact with the FP will occur either in person or by phone within 48 h of referral. The FP will describe the study, obtain consent, administer baseline assessments, and determine a primary family-centered goal. The family will then be randomized to a study condition through a centralized randomization generator.

#### Development of a service plan

In consultation with the primary care team and the behavioral health clinician, the FP will work with the family to develop a plan for services, which may include onsite integrated behavioral health services, school-based services, and/or referral to an external behavioral health clinician or agency.

#### Linkage with services

The family will receive assistance with referral and care coordination based on the child’s needs and family interest. The FP will ensure referrals are made and scheduled, and that barriers are explored through activities such as text reminders and transportation assistance.

#### Engagement with FP

Families’ ongoing FP engagement will be guided by the core components of FN. The FP will document all activities and contacts within the electronic health record (EHR). We expect that the range of activities might include assistance in obtaining school evaluations; linkage to community-based supports such as parent groups; troubleshooting challenges to accessing services; and coordinating services between primary care, school, and specialty services.

### Study conditions: Family Navigation delivery components

**Fig. 3:**
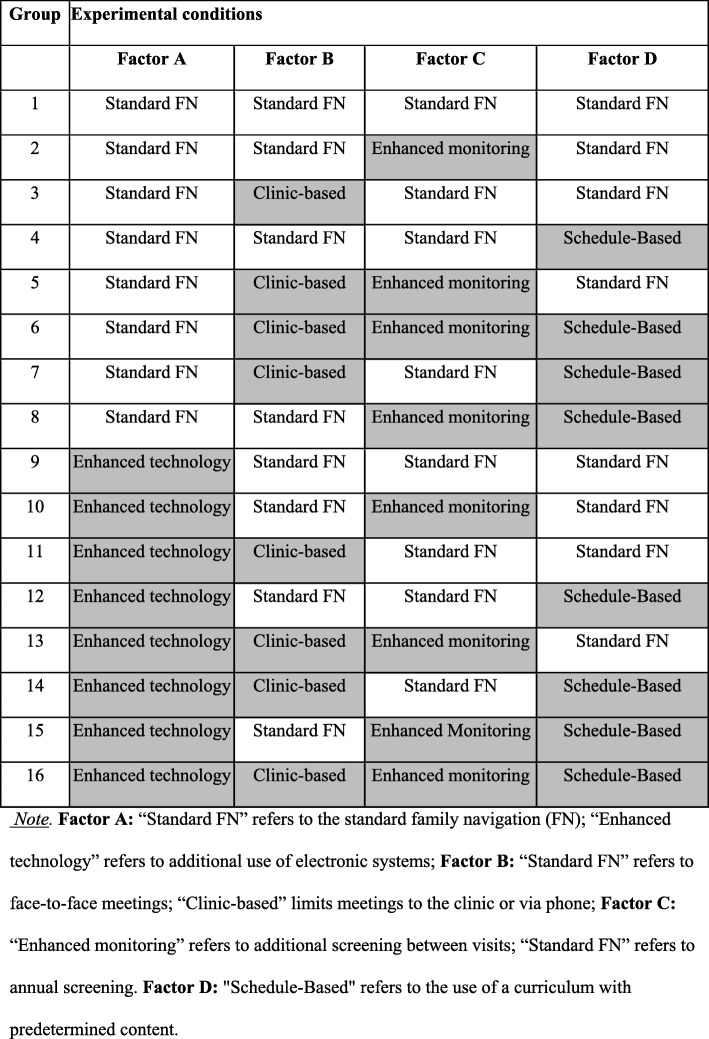
Full factorial experimental design testing four delivery strategies (4^2^: 2 × 2 × 2 × 2)

We will test four delivery strategies using a factorial design (see Fig. 3). Families will be randomized to one of 16 combinations of delivery strategies (factors): Strategy A, *Care Coordination* (i.e., usual care vs. enhanced: technology assisted); Strategy B, *Location* (i.e., clinic-based vs. clinic + community); Strategy C, *Symptom Tracking* (i.e., pediatric surveillance at annual well-child visit vs. enhanced: tracking at 3, 6, 9, and 12 months); Strategy D, *Visit Structure* (i.e., individually tailored visits vs. structured, schedule-based visits). The specific FN delivery components are described in the following sections.. Regardless of the combination of delivery strategies (core or enhanced strategies), families will all be provided the core FN intervention.

#### Strategy A: standard FN vs. enhanced: technology assisted

In core FN, FPs keep records and communicate with families using telephones and EHRs. In the enhanced condition, FPs will also have access to a cloud-based care coordination and communication software that offers administration of online questions, videoconferencing, and portals that can be used by parents and providers (e.g., FP, pediatrician, teacher). Families randomized to have access to care coordination software will work with the FP to become familiar with the features. The FP will introduce this technology to relevant school staff, e.g., the child’s teacher.

#### Strategy B: clinic-based vs. enhanced: clinic + community

In core FN (clinic-based), FPs are restricted to working at the clinic. Interactions will occur in person at the clinic and remotely via phone, text message, or other communication software. In the enhanced condition, FPs will be available to meet families in their homes and community, in addition to the clinic visits, and they will accompany families to community-based meetings. While out-of-clinic visits may substantially increase costs due to the FP’s travel (time and mileage), we hypothesize it will also improve engagement with services.

#### Strategy C: standard pediatric symptom surveillance vs. enhanced symptom tracking at 3, 6, 9, and 12 months

In core FN (surveillance at well-child visits), monitoring is determined by standard pediatric practice (annually). In experimental conditions with “enhanced monitoring,” FPs will monitor symptoms using validated instruments quarterly (the tools differ based on age) and communicate results to the child’s care team.

#### Strategy D: schedule-based vs. flexible meeting schedule

In core FN, FPs provide content at their own discretion based on perceived family needs, there is no pre-determined structure for meetings, and FPs may meet with families on an as-needed basis. In experimental conditions with scheduled visits, FPs will be expected to follow a curriculum that includes monthly meetings covering relevant topics.

### Subject allocation procedures

Before initiation of FN, FPs will assign each family an experimental condition using a computer program. The computer program will use both a randomly generated number and “minimization procedures” to minimize imbalances across conditions with respect to target variables, including family/child characteristics (e.g., gender, race/ethnicity, language). In this procedure, the first participant is assigned at random. Subsequent participants have a *p* chance of being randomly assigned and a 1 – *p* chance of being automatically assigned to the condition that would most reduce imbalance based on selected sample characteristics. Minimization procedures are considered best practices for sequential assignment [40–43]. Randomization across four binary factors results in 16 possible combinations (see Fig. 3). We plan to enroll 304 families: *n* = 38 for each cell. Unblinding of the participants will not be necessary because of the open-label nature of the trial.

### Outcomes

**Fig. 4:**
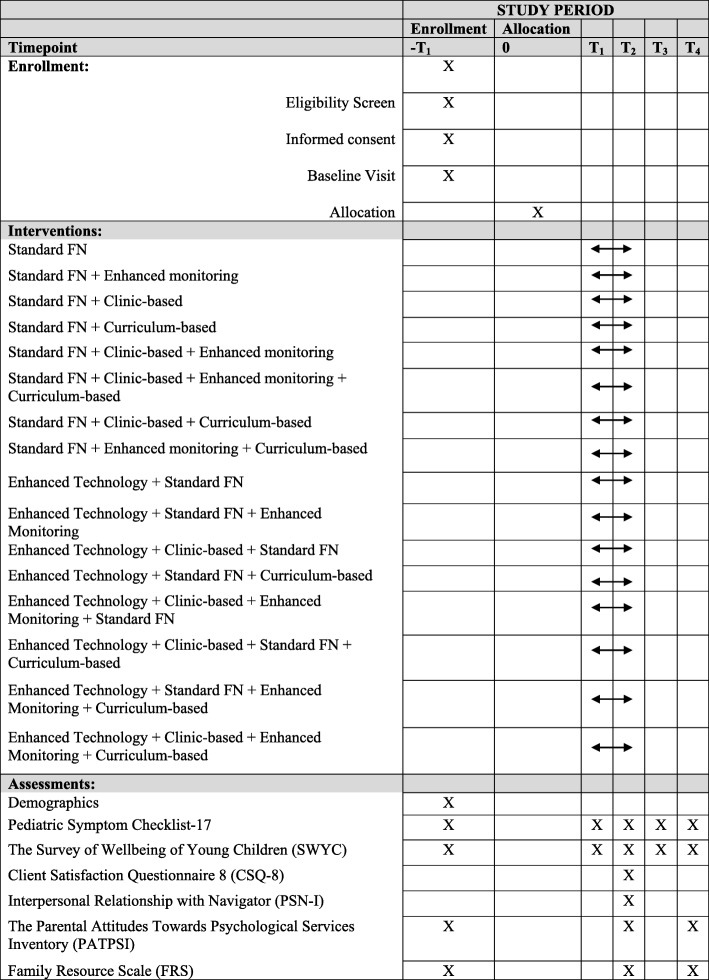
Study timetable

All children will be followed for 12 months after enrollment. Measures will be collected at enrollment and at 3, 6, 9, and 12 months. The primary method of data collection is electronic parent questionnaires administered through the EHR. As needed, such as when parents are not able to read questionnaire items, FPs will be available to assist parents in their completion of the forms. For fidelity checks and outcome data collected through medical record review, we will use blinded outcome assessors. See Fig. 4 for a timetable of the study’s enrollment schedule, interventions, assessments, and visits for participants.

#### Primary measures

The study’s primary objective, access to behavioral health services, will be measured as completion of the primary family-centered behavioral health goal outlined in the Family Plan within 90 days (yes/no). For example, for families who set a goal related to engaging in behavioral health treatment, completing the primary goal will be defined as attending a behavioral health appointment within 90 days of randomization. Related to this primary objective, we will evaluate *time to receipt of behavioral health services*, defined as time from randomization to receipt of primary behavioral health service. Dates will be obtained from administrative and billing data (EHR) for services within the recruitment health center site and FP documentation for services outside the health center. Additional engagement and child-level outcomes are described in the following paragraphs. See Table 1 for details on each outcome defined across five elements including domain, specific measurement, metric, method of aggregation, and time point.

**Table 1:** Outcome definitions across five elements

Domain	Specific measurement	Specific metric	Method of aggregation	Time points
Health services utilization	EHR record review of encounter for behavioral health service need	Time to event: total number of days between randomization and first service encounter related to behavioral health need	Proportion with an event	Continuous days from –T_1_ to T_2_
Engagement in behavioral healthcare	Documentation of FP visit dates and EHR record review of behavioral health service appointment dates and provider recommendations	≥ 4 visits with a behavioral health provider within 90 days of first FP visit, or resolution of service need as determined by behavioral health provider	Mean	T_1_
Child functioning (children aged 5.5 years and older)^a^	Pediatric Symptom Checklist-17	Total score summed from 17 items	Proportion above clinical cutoff	–T_1,_ T_4_
Child functioning (children under 5.5 years of age)^a^	The Survey of Well-being of Young Children (SWYC) Preschool Pediatric Symptom Checklist	Total score summed from 18 items	Proportion above clinical cutoff	–T_1,_ T_4_
Satisfaction with health services	Client Satisfaction Questionnaire 8 (CSQ-8)	Total score summed from 8 items	Mean	T_2_
Interpersonal relationship with Family Partner	Interpersonal Relationship with Navigator (PSN-I)	Total score summed from 9 items	Mean	T_2_
Parent attitudes	Parental Attitudes Toward Psychological Services Inventory (PATPSI)	Total score summed from 21 items	Mean	–T_1,_ T_2,_ T_4_
Parental mental health^a^	Patient Health Questionnaire-2 (PHQ-2)	Total score summed from 2 items	Proportion above clinical cutoff	–T_1,_ T_4_
Access to community resources	Family Resource Scale (FRS)	Total score summed from 30 items	Mean	–T_1,_ T_2,_ T_4_
Structural barriers	Tool for Health and Resilience in Vulnerable Environments (THRIVE) survey	Total number of social needs reported across 8 possible areas	Mean	–T_1,_ T_2,_ T_4_
Service use	EHR record review	Among scheduled visits, percentage of visits that were completed (i.e., show rate)	Mean percentage	Monthly reports

*–T*_*1*_ enrollment, *T*_*1*_ 3 months post enrollment, *T*_*2*_ 6 months post enrollment, *T*_*3*_ 9 months post enrollment, *T*_*4*_ 12 months post -enrollment

^a^Families randomized to the enhanced symptom tracking condition will receive this measure at –T_1,_ T_1,_ T_2,_ T_3,_ and T_4_

*Engagement in care* will be defined as ≥ 4 visits with a behavioral health provider within 90 days of first FP visit, or resolution of service need as determined by behavioral health provider [44].

*Child functioning* will be measured depending on the child’s age. For children aged 6years and older, the Pediatric Symptom Checklist-17 (PSC-17) will be used as a broad measure of child functioning. The PSC-17 is a 17-item psychosocial screen designed to recognize cognitive, emotional, and behavioral problems. Three subscales, Internalizing, Attention, and Externalizing, have specific cutoffs and provide additional guidance regarding need for further follow-up. The PSC-17 is embedded in the Epic (EHR) as a self-scoring form. It is widely used and has been validated in diverse populations [36–39]. For children under 5 ½ years of age, the Survey of Well-being of Young Children (SWYC) will be used to measure child functioning*.* The SWYC [35] screens for cognitive, motor, language, and social-emotional development among children up to 5½ years of age. We will track symptoms using the SWYC’s Preschool Pediatric Symptom Checklist (PPSC) [34], an 18-item questionnaire that has demonstrated strong validity and acceptability in diverse populations. Translations are available in a range of languages.

#### Secondary measures

Secondary patient experience outcomes will be measured at baseline and at 3, 6, 9, and/or 12 months. Secondary outcome measures will be used to evaluate the theory-based mechanisms of FN effectiveness and indicate for whom the FN delivery strategy is most effective (i.e., assessment of mediators and moderators). Theory-based mechanisms are both related to the *person* (e.g., attitudes) and the *system* (i.e., access to resources, overcome barriers). See Table 2 for the specific intervention targets and theoretical foundations.

**Table 2:** Theory-based mechanisms and measures

Domain	Target	Theoretical mechanism	Instrument
Person	*Parent experience*	Improved parental attitudes about mental health increases capacity to engage in services	PATPSI
	*Parent experience*	Improved mental health increases capacity to engage in services	Patient Health Questionnaire-2
System	*Access to resources*	Improving social determinants increases access to resources	Family Resource Scale
	*Structural barriers*	Coordination decreases structural barriers	EHR FP templates

*Satisfaction with health services* will be with measured with the Client Satisfaction Questionnaire 8 (CSQ-8); i.e., it will be used to assess family satisfaction with health services. This measure has established psychometric properties with ethnically diverse populations [45–47].

*Interpersonal relationship with FP* will be measured using the Interpersonal Relationship with Navigator (PSN-I). This measure is a validated 9-item scale with strong psychometric properties in samples of culturally diverse, underserved patients [48].

To assess *parent attitudes*, we will use the Parental Attitudes Toward Psychological Services Inventory (PATPSI). This measure consists of 21 Likert-type items (0 = strongly disagree; 5 = strongly agree) that assess parents’ help-seeking attitudes, help-seeking intentions, and mental health stigma [49].

To assess *parental mental health*, the Patient Health Questionnaire-2 (PHQ-2) [50, 51] will be administered. The PHQ-2 is a validated two-question depression screener with score ranges from 0 to 6. The authors of the measure identified a score of 3 as the optimal cut point when using the PHQ-2 to screen for depression. If the score is 3 or greater, major depressive disorder is likely.

*Access to community resources* of families will be measured with the Family Resource Scale (FRS) [52, 53], a 30-item scale that assesses family concerns regarding adequacy of resources. We hypothesize that FN will improve *access to resources* over time. The FRS assesses numerous family needs and factors including growth/support, health/necessities, physical necessities, physical shelter, intra-family support, communication/employment, child care, and personal resources. It is a self-administered instrument; each item is rated on a 5-point scale ranging from “not at all adequate” to “almost always adequate.”

*Structural barriers* will be alleviated with the collection of monthly *professional contact data*, using FN logs on contacts with others on the care team. We will measure level of care coordination (number and frequency of contacts between care providers) and existence of barriers as delineated in the logs. Additionally, Tool for Health and Resilience in Vulnerable Environments (THRIVE) data will be collected as part of usual care and recorded in the EHR [54]. THRIVE is a social determinant of health survey based on parent-reported need across eight domains: housing, food, utilities, paying for medications, caregiving, transportation, payment for medical employment, and education.

Data regarding *service use* will be collected through the EHR. This will include the FP’s documentation and show rate (i.e., appointments scheduled and completed).

#### Implementation measures

For each delivery strategy, we will collect data on fidelity, acceptability, feasibility, and cost.

##### Fidelity to the FN core model and delivery strategies

We will use multiple data sources to assess fidelity using methods employed in prior studies [16–19, 25–27, 29]. We will review structured navigation visit templates integrated into the EHR and FP contact logs monthly. A random sample of two visits per month will be reviewed using a Navigation Checklist we developed for “real-time” monitoring. MI will be assessed quarterly using audiotaped standardized patient interactions, which will be scored using the Motivational Interviewing Supervision and Training Scale (MISTS) [55, 56]. We will assess both fidelity to the core model and fidelity to the delivery strategies; for example, we will check if FPs are administering symptom monitoring, and how frequently, in the “symptom tracking” condition. If an FP is not meeting fidelity criteria, she will be provided with retraining and additional support. Once per year, the FPs will participate in an MI booster training session to maintain their MI skills.

##### Acceptability and feasibility

We will use qualitative methods to assess acceptability of each delivery strategy. We will use purposeful sampling to interview five subjects from each of the 16 strategies (*n* = 80). Interview questions will be based on the CFIR. Because the goal of the study is delivery optimization, we will specifically focus our questions on the eight domains that comprise the “Intervention Characteristics” construct within the CFIR: source, evidence strength and quality, relative advantage, adaptability, trialability, complexity, design quality and packaging, and cost.

##### Cost

We will use *time-driven activity-based costing (TDABC)* to develop cost estimates for each delivery strategy [57]. TDABC’s goal is to develop valid estimates of service costs while minimizing expenditures on research [57]. It accomplishes this goal by requiring only two key sets of estimates: *capacity cost rate* and *demand for resource capacity*. Because the explicit goal of using TDABC in our project is to support process optimization and enhance scalability, we will not calculate costs over the entire care delivery value chain [58]. Instead, we focus only on specific FN activities, consistent with the use of TDABC in studies of healthcare processes [59].

We will first calculate the capacity cost rate for the FP as a function of total annual compensation divided by the time available for FN activities. Next, we will estimate the demand for resource capacity resulting from each FN delivery strategy. Assumptions regarding FN activities that create demand for resource capacity will be based on process maps [59]. Process maps developed during a previous study of FN will be adapted and refined to reflect FN procedures in the current project [60]. Based on these process maps, staff reports, and direct observations, we will develop time equations for each FN delivery strategy. Such time equations closely resemble linear regression equations in that they include an intercept that reflects a baseline time estimate, as well as coefficients and dummy variables that reflect additional time required to account for variations. For example, a time equation for an FN phone call to a patient might include an intercept of 2 min to look up a phone number and make a call, a coefficient that adds 5 min if the patient answers, and an additional 10 min if a survey is administered.

### Data management

All participants will be assigned a unique study code. This study code will be used to link data from the EHR, billing records, and Act.MD. The crosswalk that links study codes to participant names will be kept in a locked office and separate from the data. This crosswalk will be kept for the duration of the study and then destroyed. Any paper surveys utilized will be transferred the same day that they are completed to a locked file cabinet in the locked office of a study Principal Investigator.

All collected data will be stored on a Health Insurance Portability and Accountability Act (HIPAA)-compliant, password-protected, secure drive maintained by the institutions sponsoring the study. The study utilizes data collection and management tools that meet HIPAA security rules to protect the confidentiality and security of protected health information.

### Statistical analyses

#### Overview

All statistical analyses will be done in SAS (v9.4) and MPlus (v8). Baseline characteristics of parents and children will be compared across conditions to assess balanced randomization. Characteristics include race/ethnicity, insurance status, primary language, and child characteristics (e.g., age).

#### Main effects

Following an intent-to-treat model, multiple regression models will be used to test hypotheses regarding the main effects of the four delivery strategies and their combined effects on the study’s primary outcome. A series of increasingly complex models will be constructed to address each specific index of outcome. For example, a logistic regression analysis will test receipt of behavioral health services within 90 days. Cox regression (proportional hazards) analyses will then be used to test the effect of each factor on time to receipt of services. Similarly, engagement in services will first be analyzed with logistic regression using our definition of engagement in care as a binary outcome, with subsequent multilevel models to analyze engagement in multiple services.

We will examine all data for missing information and loss to follow-up. We plan to consult with a statistician regarding any missing data and will use multiple imputation as appropriate. For outcomes involving engagement, sensitivity analyses will be conducted in which missing data from the EHR is interpreted as failure to engage in services. While we do not hypothesize interactions among the delivery strategies, these will also be explored. Following recommendations for factorial designs, effect coding (not dummy coding) will be used for experimental conditions to assess for interaction. In addition to evaluating effects “at the margins” using all available cells, results for each individual cell will also be reported [61], as will simple main effects.

#### Mediator/moderator analyses to examine intervention mechanism

Consistent with our theoretical model and based on our prior studies and literature review, we hypothesize that FN intervention effects will be mediated by parents’ capacity to pursue services, access to services, and structural barriers. We will examine mediational effects using two different, but related, methods: the approach of Baron and Kenny and the use of path analysis models. Each approach can be used to differentiate between direct and indirect intervention effects. In the path analysis models (which have greater statistical power), we will create a series of nested models based on our theoretical model in which we will systematically vary model parameters and constraints to test the effect of each potential mediator. Nested models will be compared using difference tests and other standard indices (Akaike’s information criterion, the comparative fit index (optimal value > 0.95), the Tucker-Lewis index (optimal value > 0.95), and the root mean square error of approximation (optimal values < 0.06)). We will fit these models with MPlus software, which allows for the modeling of continuous and dichotomous, endogenous, and exogenous variables. While our study design only allows for direct testing of the causal effects of primary delivery strategies A, B, C, the causal effect of mediating variables can be analyzed by treating factors as instrumental variables in the path analysis [62, 63].

#### Moderator analyses

We will evaluate the extent to which each delivery strategy, race/ethnicity, primary language, and symptom severity moderate FN effects using stratified analysis. Previous studies have found no effect of such demographic variables on the effect of FN. We hypothesize that any effects will be small and clinically non-significant; however, we will perform these analyses, as evaluation of moderators is important to ensure equity.

### Sample size and power

Of the three measures to operationalize engagement, we powered our study on the dichotomous variable “achieved goal related to receipt of mental health services within 90 days” (the most conservative estimate). We based our power calculation on the number needed to detect the smallest differences in primary outcomes that are of clinical importance. Our formative work with staff at the recruitment health center and other community health centers indicated that a relative risk of approximately 25% would be considered clinically significant. Therefore, if 60% of families in the core FN condition engage in mental health services (estimates based on our prior work), in order to detect a 25% difference (i.e., 75% of families in any of the FN delivery conditions engage in services) and assuming two-tailed tests and a type 1 error rate of 5%, approximately 304 participants are required to detect this effect (*n* = 19 in each of 16 cells).

We expect strong effects of study mediators, in particular fidelity variables and variables that are central to our theoretical model, such as increased parent capacity. We estimate that our design will have at least 80% power to detect mediation effects where the paths from independent variable to mediator and from mediator to outcome are of at least small-to-medium Effect Size (ES = .26). Given that our mediation analyses are designed to support decisions regarding intermediate outcomes to be tracked for quality control and assurance, effect sizes less than this magnitude are not considered to be clinically important. In contrast, analyses of patient-level treatment moderators are exploratory, as we have no evidence to support hypotheses of any effect.

### Qualitative data analysis

Each interview will be independently coded by two members of the research team, using the CFIR codebook. The interviews will then be collectively reviewed to ensure coding consensus and reconcile discrepancies. A review of all of the codes for each interview will be conducted until members of the research team reach consensus as to which codes should be applied to specific segments of text. After consensus is achieved among coders, interview transcripts will then be entered, coded, and analyzed in QSR-NVivo.

### Final evaluation

The final stage of the MOST framework—Evaluation—will be conducted after completing data analysis. We will convene key stakeholders in behavioral health, FN, and policy to develop consensus recommendations regarding FN delivery. Data on effectiveness of each component both in isolation and in combination, secondary outcomes, and implementation will be presented to our stakeholder panel. Then, a modified Delphi approach [64] will be used to select components for inclusion in the final intervention package.

### Post-trial care

Access to FN through the study will end with the termination of the trial. However, participants will be able to continue accessing care within and outside of the federally qualified community health center, whether or not the FP played a role in supporting access to that care. In the absence of FPs available to support families through study participation, families will follow general clinical protocols for behavioral health case management and referral.

## Discussion

In the current study we use the MOST framework to optimize FN delivery. Using a factorial design, we will develop an optimized, efficient, effective version of FN. The goal of the intervention is to improve access to, and engagement in, diagnostic and treatment services for children with behavioral health disorders. This approach represents important advances in the field of implementation science for several reasons.

First, we are using the MOST framework to optimize intervention delivery. While MOST is a framework for optimization, it is important to note that what one optimizes on (e.g., clinical outcomes, implementation, cost) is determined by the key stakeholders involved in the study. Thus, in this study we are specifically using MOST as a framework for *delivery*. This study protocol can serve as a guide for others working to optimize intervention delivery. The framework holds a unique benefit in that the majority of methodologies used for optimization do not include the rigor of randomization. Thus, understanding these methods can be of great value to the field.

Second, these methods can help inform others looking to optimize an intervention designed to alleviate disparities in access to services. Low-income and ethnically diverse children with mental/behavioral health concerns often experience delays in obtaining a diagnosis and appropriate evidence-based treatments [65, 66]. Solutions to mitigate these disparities, such as FN, must be delivered in an efficient and effective manner to increase the likelihood of sustainable, widespread adoption. Furthermore, FN has been implemented in real-world practice with different strategies, and with varying success [26–28]. The current study will advance our understanding of which delivery strategies are the most effective, and for whom. Given that some delivery strategies are more labor- and resource-intensive than others, to be efficient, FN programs understand “active ingredients” that lead to the most positive outcomes while leaving out potentially expensive and time-consuming strategies with lesser impact.

Finally, current health service delivery reforms are promoting primary care networks as the ”hub” of care coordination. Financial incentives created under the Affordable Care Act are spawning new systems that link primary care and specialty services within integrated networks. For example, Accountable Care Organizations (ACOs) [67] are groups of doctors, hospitals, and other healthcare providers who join together to give coordinated, high-quality care. Implementation of FN within the setting of a newly formed ACO (Boston Accountable Care Organization) links this innovation to the broader policy context and maximizes scale-up potential within emerging delivery systems. Understanding delivery within this new context is important to FN’s ultimate success.

### Challenges and potential solutions

The two main challenges we anticipate are related to the complexity of subject assignment and the pragmatic nature of the study. Specifically, because we are using the MOST framework and a factorial design, we will be randomizing families to one of 16 conditions. There is therefore a large burden on the study team to prevent contamination across conditions, and monitoring for fidelity of each condition. Contamination is a universal concern for studies using the MOST framework [68]. We have implemented several strategies to prevent contamination. First, we are using an electronic randomization protocol that allows the Navigators to directly randomize to a condition in real time. This allows Navigators to know, at the time of enrollment, which of the 16 conditions a family is randomized to. This program also allows us to input which condition the family is assigned into the medical record. Keeping this data within the medical record helps ensure that whenever a FP is working with the family, she is immediately alerted as to which condition the family is assigned. Finally, each condition is given a “code name” to support accurate categorization of each family into their correct condition. This code name is documented in the medical record as well.

The second challenge relates to the fact that this is a pragmatic trial embedded within usual care of a large, federally qualified health center, and we may lose the ability to tightly control the use of each condition. For example, if a Navigator or families choose not to use the web-based care coordination software, we will not have the ability to ensure use. Although this may make assessment of outcome data difficult (i.e., effectiveness of a condition that is insufficiently used), it does allow us to better understand how conditions may be used in the “real world.”

### Trial status

The study start date was June 24, 2019. The current protocol version date is June 26, 2019. The protocol contributors were Emily Feinberg (Co-Principal Investigator), Lisa Fortuna (Co-Principal Investigator), Sarabeth Broder-Fingert (Co-Investigator), Radley Christopher Sheldrick (Co-Investigator), Megan Jordan (Co-Investigator), Dana Rubin (Co-Investigator), and Andrea Chu (Project Manager).

## Conclusions

This study uses the MOST framework to optimize FN delivery. We are comparing a specific delivery package against the aggregate effect of all the other delivery strategies. This framework offers a more efficient strategy for testing multiple delivery strategies over a traditional multiarm trial. These methods will be useful for future investigators working to optimize interventions for implementation and dissemination.

## Supplementary information

**Additional file 1.** World Health Organization Trial Registration Data Set.

**Additional file 2.** SPIRIT 2013 checklist: recommended items to address in a clinical trial protocol and related documents.

## Data Availability

The study investigators will have access to and manage the final study dataset. Data collected as part of this study will be submitted to the National Database for Clinical Trials Related to Mental Illness (NDCT) per the data-sharing guidelines of the National Institute of Mental Health (NIMH) (NOT-MH-14-015) to be shared with other investigators.

## Abbreviations

ACO: Accountable Care Organization
ADHD: Attention deficit/hyperactivity disorder
ASD: Autism spectrum disorder
EHR: Electronic health record
FN: Family Navigation
FP: Family Partner
FRS: Family Resource Scale
MI: Motivational interviewing
MISTS: Motivational Interviewing Supervision and Training Scale
MOST: Multiphase Optimization Strategy
PHQ-2: Patient Health Questionnaire-2
PPSC: Preschool Pediatric Symptom Checklist
PSC-17: Pediatric Symptom Checklist-17
PSN-I: Interpersonal Relationship with Navigator
SWYC: Survey of Well-being of Young Children (SWYC)
