# Supplementary material for: Using the Multiphase Optimization Strategy (MOST) framework to test intervention delivery strategies: a study protocol
Source: Trials. 2019 Dec 16;20:728. doi: 10.1186/s13063-019-3853-y (PMC6915979; doi:10.1186/s13063-019-3853-y)
Supplement: Supplementary file 1 — Additional file 1. World Health Organization Trial Registration Data Set. [file 13063_2019_3853_MOESM1_ESM.docx]

World Health Organization Trial Registration Data Set

| **Data category** | **Information** |
| --- | --- |
| Primary registry and trial identifying number | ClinicalTrials.gov NCT03569449 |
| Date of registration in primary registry | June 26, 2018 |
| Secondary identifying numbers | H-37634 |
| Source(s) of monetary or material support | National Institute of Mental Health (NIMH) |
| Primary sponsor | National Institute of Mental Health (NIMH) |
| Secondary sponsor(s) | NA |
| Contact for public queries | Emily Feinberg, ScD, CPNP, Boston Medical Center and Boston University School of Public Health, Boston, MA |
| Contact for scientific queries | Emily Feinberg, ScD, CPNP, Boston Medical Center and Boston University School of Public Health, Boston, MA |
| Public title | Optimizing a Paraprofessional, Family Navigation Model for Children |
| Scientific title | Optimizing a Paraprofessional, Family Navigation Model for Children |
| Countries of recruitment | United States |
| Health condition(s) or problem(s) studied | Health Behavior |
| Intervention(s) | All families will work with a family partner. Families will be randomly assigned to one of 16 different ways that a Family Partner can work with a family:   1. Clinic-based visit, usual care, standard pediatric surveillance, and structured visits 2. Clinic-based visit, usual care, enhanced pediatric surveillance, and structured visits 3. Clinic-based visit, technology-enhanced care coordination, standard pediatric surveillance, and structured visits 4. Clinic-based visit, technology-enhanced care coordination, enhanced pediatric surveillance, and structured visits 5. Clinic-based visit, usual care, standard pediatric surveillance, and individually-tailored visits 6. Clinic-based visit, usual care, enhanced pediatric surveillance, and individually-tailored visits 7. Clinic-based visit, technology-enhanced care coordination, standard pediatric surveillance, and individually-tailored visits 8. Clinic-based visit, technology-enhanced care coordination, enhanced pediatric surveillance, and individually-tailored visits 9. Clinic and community visits, usual care coordination, standard pediatric surveillance, and structured visits 10. Clinic and community visits, usual care coordination, enhanced pediatric surveillance, and structured visits 11. Clinic and community visits, technology enhanced care coordination, standard pediatric surveillance, and structured visits 12. Clinic and community visits, technology enhanced care coordination, enhanced pediatric surveillance, and structured visits 13. Clinic and community visits, usual care coordination, standard pediatric surveillance, and individually-tailored visits 14. Clinic and community visits, usual care coordination, enhanced pediatric surveillance, and individually-tailored visits 15. Clinic and community visits, technology-enhanced care, standard pediatric surveillance, and individually-tailored visits 16. Clinic and community visits, technology-enhanced care, enhanced pediatric surveillance, and individually-tailored visits |
| Key inclusion and exclusion criteria | Inclusion Criteria:  All children who are 3-12 years old:   - who screen positive on the Survey of Wellbeing of Young Children (SWYC) (3-5 years) OR - who screen positive on the Pediatric Symptom Checklist-17 (PSC-17) (6-12 years) OR - whose parents indicate a behavioral health concern during any pediatric visit   Exclusion Criteria:   - Children who are already actively engaged in behavioral health specialty care services, defined as having had a behavioral health visit in the last 30 days, who do not require new additional services - Children with active psychosis - Children with safety concerns requiring emergency mental health services. |
| Study type | Allocation: Randomized Intervention Model: Factorial Assignment Masking: Single (Investigator) Primary Purpose: Health Services Research |
| Date of first enrolment | June 24, 2019 |
| Target sample size | 304 |
| Recruitment status | Recruiting |
| Primary outcome(s) | - Time to services - Access to services - Change in the Survey of Well-being of Young Children (SWYC) - Change in Pediatric Symptom Checklist-17 (PSC-17) |
| Key secondary outcomes | - Retention in services - Client Satisfaction Questionnaire - Interpersonal Relationship with Navigator (PSN-I) - Change in Family Resource Scale (FRS) - Change in Patient Health Questionnaire- (PHQ-2) - Change in Parental Attitudes Towards Psychological Services Inventory (PATPSI) |
